# Supplementary material for: Aminoglycoside-Modifying Enzymes Are Sufficient to Make Pseudomonas aeruginosa Clinically Resistant to Key Antibiotics
Source: Antibiotics (Basel). 2022 Jul 1;11(7):884. doi: 10.3390/antibiotics11070884 (PMC9312099; doi:10.3390/antibiotics11070884)
Supplement: Supplementary file 1 [file antibiotics-11-00884-s001.zip › antibiotics-1756798-Tables S2, S3, S4, S5, S6.pdf]

Supporting material

Aminoglycoside-modifying enzymes are sufficient to make *Pseudomonas aeruginosa* clinically resistant to key antibiotics

Authors: A. Thacharodi and I. L. Lamont.

Table S2: Aminoglycoside-modifying enzymes identified in this study

Table S3: Transposable elements in *P. aeruginosa* isolates analysed in this study

Table S4: MICs of *P. aeruginosa* containing pSW196

Table S5: Bacterial isolates and plasmids used in this study

Table S6: Primers used in this study

Table S2. Aminoglycoside modifying enzymes identified in this study[a]

	Enzymes	Genetic location	Accession Number	Resistance[b]	Prevalence %[b]			Comments	Isolates contain AMEs	Ref
					C	P	W			
					Aminoglycoside N-acetyltransferase					
**1**	Aac(2')-IIa	Plasmid, integron	AB669090	KSM	0.38	0.0	0.02	Found in *B. glumae* and *A. avenae*	PAC14B	[1]
**2**	Aac(3)-I	Integron, transposon	AJ877225	G	-	-	-	Reported in integron cassette of *P. aeruginosa*	A106	[2,3]
**3**	Aac(3)-Ia	Plasmid, transposon, integron	X15852 AF550679	G	0.0	0.0	0.88	Found in *P. aeruginosa*, *E. coli*, *K. pneumoniae* and *A. baumannii*	EC2A, 403-104	[4]
**4**	Aac(3)-IIa	Plasmid	X13543	T, G	0.0	0.0	0.02	Reported in *P. aeruginosa* and *E. coli*	197S020911BSL_PA3	[5]
**5**	Aac(3)-IIIc	Not known	L06161	T, G, K, N, P	0.0	0.0	0.02	Reported in P. *aeruginosa.* Rarely reported in *Enterobacteriaceae*	AUS106, 403-102	[6]
**6**	Aac(3)-IV	Plasmid	X01385	T, G	0.0	0.0	0.04	Binds to apramycin and gentamicin, Commonly found in *Salmonella spp*.	6102, 6000.3	[7]
**7**	Aac(6')-Ib3 (aacA4)	Integron	X60321	T, N, K, G	0.0	0.0	0.33	AAC (6′)-Ib has been described in a few CF patient isolates resistant to tobramycin	HCF73, 1257147, 1260990, 1275655, 1344658, 1420275, 1690076, 1295835, 1586994	[8]
**8**	Aac(6')-II (aacA7)	Plasmid, integron	U13880	T, G	0.0	1.92	2.08	Aac(6′)-II enzymes catalyze acetylation	1301482, 1324459, 1586981	[9]

								of all forms of gentamicin but not of amikacin<br><br>Observed in *P. aeruginosa*		
**9**	Aac(6')-IIa	Plasmid, Integron	M29695	T, G	0.0	0.0	0.35	Common among *P. aeruginosa* and *S. enterica*	5994	[10]
**10**	Aac(6')-31	Integron	AM283348	A, T, G, K, N, I	0.0	0.0	0.71	Observed in *P. putida*, *A. baumannii* and *K. pneumoniae*	1586981	[11]
**11**	Aac(6')-33	Integron	GQ337064	A, T	0.0	0.0	0.20	Observed in *P. aeruginosa*	1275655, 1420275, 1690076	[12]
Aminoglycoside O-nucleotydyltransferases										
**12**	Ant (2")-Ia (aadB)	Plasmid, Integron	X04555	T, G, K	3.07	5.77	4.82	Found in gentamicin-resistant and tobramycin-resistant clinical isolates of *P. aeruginosa.* Widespread among all gram-negative bacteria	2D9A, 5999.1 5999.2 5999.3 318S170811BSL_PA1 298S020611BSL_PA1 278S180511BSL_PA2 278S180511BSL_PA1 66S100212BSL__PA1 008A1, A106, 403-105, 1268230 1271701, 1257147, 1324459, 1607533, AUS720	[5]
**13**	AadA1 (Ant(3")-Ia)	Plasmid, Integron, Transposon	X02340	S	3.07	1.92	1.7	Found among Enterobacteriaceae, *A. baumannii*, *P. aeruginosa* and *Vibrio cholerae*	1271701, 1324459	[13]

**14**	AadA1b (Ant(3"))	Plasmid, Integron, Transposon	M95287	S	3.94	1.03	1.74	Common in *Enterobacteriaceae, A. baumannii*, *P. aeruginosa* and *V. cholerae.*	1275655, 1420275,1690076	[14]
**15**	AadA2 (Ant(3"))	Plasmid, integron	NC_010870	S	0.0	0.0	1.06	Common in *K. pneumoniae* and *Salmonella spp.*	318S170811BSL_PA1	[14]
**16**	AadA2b (Ant(3"))	Plasmid, Integron	D43625	S	0.0	0.0	1.0	Located on integron InC, Isolated from the R-plasmid of *P. aeruginosa*, as a gene cassette	5999.1 5999.2 5994, 5999.3 66S100212BSL__PA1, 1344658	[15]
**17**	AadA4 (Ant(3"))	Plasmid, Chromosome	NC_002928 NC_010558	S	0.38	0.0	0.07	Found in *Bordetella parapertussis* and *E. coli*	A106	[16]
**18**	AadA6 (Ant(3"))	Integron	AM087411	S	1.53	0.0	4.15	Found encoded in the integron of *P. aeruginosa*	SMC1587, 4064320487 272S250511BSL_PA1 1260990, 1586981, 1586994, 5014375233	[17]
**19**	AadD (Ant(4')-Ia)	Plasmid	U35229	A, T, K, N	Not available			Common among *S. epidermidis*, *S. aureus*, *Enterococcus spp*. and *Bacillus spp*	6099	[18]
					**Aminoglycoside O-phosphotransferases**					
**20**	Aph(3")-Ib	Plasmid, Transposons, chromosomes, IC elements	M28829	S	0.77	0.0	0.22	Found in *P. aeruginosa* and *E. coli*	3C52, AT31, HCF73, 5, 298S020611BSL_PA1 278S180511BSL_PA2 286S270711BSL_PA1 278S180511BSL_PA1 66S100212BSL__PA1	[19]

									C54, 1313352, 1257147, 1344658, 1607533	
**21**	Aph(3')-IIa (AphA-2)	Transposon	V00618	K, N, P, GmB	0.38	1.92	0.53	Common among *E. coli*	1275655	[20]
**22**	Aph(3')-VIa (AphA-6)	Plasmid Chromosome	X07753	A, K, N, P, GmB, I	2.31	0	0.92	Primarily isolated from *Acinetobacter spp.*<br><br>Was cloned into *E. coli* to study amikacin resistance	1260990	[21]
**23**	Aph(4)-Ia	Plasmid	V01499	HygB	0.0	0.0	0.04	Found among *E. coli*	6102, 6000.3	[22]
**24**	Aph(6)-Ic (str sph)	Transposon	X01702	S	0.38	0.0	0.24	Found in *S. enterica*, *P. aeruginosa* and *E. coli*.	1275655	
**25**	Aph(6)-Id (strB, orfI)	Plasmid, Transposons, chromosomes, IC elements,	M28829	S	4.6	11.54	4.88	APH (6)-Id is a streptomycin phosphotransferase<br><br>Found in *K. pneumoniae*, *Salmonella spp*., *E. coli*, *Shigella flexneri*, *Pseudomonas spp*., *V. cholerae.*	3C52, AT31, 5, 298S020611BSL_PA1 278S180511BSL_PA2 286S270711BSL_PA1 278S180511BSL_PA1 66S100212BSL__PA1 C54, 1313352, 1257147, 1344658, 1607533	[23]

[a] Genes encoding aminoglycoside-modifying enzymes were identified using ResFinder 4.1 [24] and confirmed with Resistance Gene Identifier (RGI) version 4.2.2 with information drawn from the CARD database 3.0.1[25].

[b] Abbreviations: Resistance: P- paromycin, I- isepamicin, A-amikacin; G-gentamicin, GmB- gentamicin B; K-kanamycin; N-neomycin; S-streptomycin; T-tobramycin, HygB-hygromycin B, KSM-kasugamycin; Prevalence %: C- NCBI Chromosome, P- NCBI Plasmid, W- NCBI WGS

Table S3. Transposable elements in clinical isolates of *P. aeruginosa*[a]

MLST	MGE	Resistance Genes	Phenotype	Accession
**Isolate 1257147**				
235	Tn6082<br>Unit Transposon	*aac(6')-Ib-cr*	ciprofloxacin, tobramycin, amikacin	EF636461
		sul1	sulfamethoxazole	U12338
		aph(3'')-Ib	streptomycin	AF024602
		aph(6)-Id	streptomycin	M28829
		dfrA5	trimethoprim	X12868
		aac(6')-Ib3	tobramycin, amikacin	X60321
		blaOXA-17	cefotaxime, ampicillin, cefepime, ceftazidime, cefixime, aztreonam, amoxicillin	DQ902344
		ant(2'')-Ia	tobramycin, gentamicin	JF826500
		qacE	chlorhexidine, benzylkonium chloride, ethidium bromide, cetylpyridinium chloride	X68232
		blaOXA-129	unknown beta-lactam	FJWZ01000025
	ISPa7<br>Composite transposon	*fosA*	fosfomycin	ACWU01000146
		cmx	chloramphenicol	U85507
	ISPa7<br>Composite transposon	*aac (6')-Ib-cr*	ciprofloxacin, tobramycin, amikacin	EF636461
		catB3	chloramphenicol	U13880
		qacE	chlorhexidine, benzylkonium chloride, ethidium bromide, cetylpyridinium chloride	X68232
		sul1	sulfamethoxazole	U12338
		blaOXA-1	ampicillin+clavulanic acid, amoxicillin+clavulanic acid, ampicillin, piperacillin+tazobactam, piperacillin, cefepime, amoxicillin	HQ170510
		ARR-3	rifampicin	JF806499
**Isolate 1260990**				
395	ISPa1635<br>Composite transposon	*fosA*	fosfomycin	ACWU01000146
		sul1	sulfamethoxazole	U12338
		qacE	ethidium bromide, chlorhexidine, benzylkonium chloride, cetylpyridinium chloride	X68232
		blaVIM-2	piperacillin, piperacillin+tazobactam, ceftazidime, cefixime, ertapenem, meropenem,	AF302086

			amoxicillin, cefepime, amoxicillin+clavulanic acid, imipenem, ampicillin, cefoxitin, ampicillin+clavulanic acid, cefotaxime	
		aph(3')-VIa	gentamicin, amikacin, butirosin, kanamycin, paromomycin, neomycin, ribostamycin	X07753
		qacE	ethidium bromide, chlorhexidine, benzylkonium chloride, cetylpyridinium chloride	X68232
		aac(6')-Ib-cr	ciprofloxacin, amikacin, tobramycin	EF636461
		aadA6	spectinomycin, streptomycin	AF140629
		aac(6')-Ib3	amikacin, tobramycin	X60321
		blaOXA-10	piperacillin, piperacillin+tazobactam, aztreonam, amoxicillin, ampicillin	J03427
		catB7	chloramphenicol	AF036933
		crpP	ciprofloxacin	HM560971
**Isolate 1275655**				
235	Tn5563	*fosA*	fosfomycin	ACWU01000146
		aph(6)-Ic	streptomycin	X01702
		aph(3')-IIa	neomycin, kanamycin	V00618
	IS6100 Composite transposon	*aac(6')-33*	tobramycin, amikacin	GQ337064
		blaGES-19	ticarcillin, ampicillin+clavulanic acid, piperacillin, cefoxitin, ampicillin, amoxicillin, amoxicillin+clavulanic acid, ceftazidime	JN596280
		qacE	benzylkonium chloride, ethidium bromide, chlorhexidine, cetylpyridinium chloride	X68232
		sul1	sulfamethoxazole	X15024
		blaGES-20	ticarcillin, ampicillin+clavulanic acid, piperacillin, cefoxitin, ampicillin, amoxicillin, amoxicillin+clavulanic acid, ceftazidime	JN596280
		aac(6')-Ib-cr	tobramycin, amikacin, ciprofloxacin,	EF636461
		blaOXA-2	ampicillin+clavulanic acid, piperacillin, ampicillin, amoxicillin, amoxicillin+clavulanic acid, ceftazidime	DQ112222
		aac(6')-Ib3	tobramycin, amikacin	X60321

		ant(2")-Ia	gentamicin, tobramycin	X04555
**Isolate 1324459**				
357	Tn4661<br>Unit Transposon	*fosA*	fosfomycin	ACWU01000146
		blaVEB-1	aztreonam, ticarcillin+clavulanic acid, cefotaxime, cefoxitin, cefepime, ticarcillin, amoxicillin, piperacillin+tazobactam, amoxicillin+clavulanic acid, ampicillin, ceftazidime, ampicillin+clavulanic acid, piperacillin	HM370393
		ant(2'')-Ia	tobramycin, gentamicin	X04555
		crpP	ciprofloxacin	HM560971
		dfrB2	trimethoprim	AY553333
		blaOXA-10	aztreonam, amoxicillin, piperacillin+tazobactam, ampicillin, piperacillin	J03427
		aac(6')-Il	amikacin, tobramycin	U13880
		tet(A)	tetracycline, doxycycline	AY196695
		qacE	benzylkonium chloride, chlorhexidine, ethidium bromide, cetylpyridinium chloride	X68232
		sul1	sulfamethoxazole	U12338
		catB7	chloramphenicol	AF036933
**Isolate 1420275**				
309	Tn5563<br>Unit transposon	*fosA*	fosfomycin	ACWU01000146
		aac(6')-33	amikacin, tobramycin	GQ337064
		qacE	benzylkonium chloride, chlorhexidine, cetylpyridinium chloride, ethidium bromide	X68232
		sul1	sulfamethoxazole	X15024
		blaGES-20	piperacillin, amoxicillin, amoxicillin+clavulanic acid, ampicillin+clavulanic acid, ampicillin, ticarcillin, ceftazidime, cefoxitin	JN596280
		aac(6')-Ib-cr	ciprofloxacin, amikacin, tobramycin	EF636461
		blaOXA-2	piperacillin, amoxicillin, amoxicillin+clavulanic acid, ampicillin+clavulanic acid, ampicillin, ceftazidime	DQ112222
		aac(6')-Ib3	amikacin, tobramycin	X60321
		ant(2")-Ia	gentamicin, tobramycin	M95287
		catB7	chloramphenicol	AF036933
		crpP	ciprofloxacin	HM560971
		blaGES-19	piperacillin, amoxicillin, amoxicillin+clavulanic acid, ampicillin+clavulanic acid,	JN596280

			ampicillin, ticarcillin, ceftazidime, cefoxitin	
**Isolate 1586981**				
235	ISPa7 Composite transposon	*fosA*	fosfomycin	ACWU01000146
		blaOXA-2	piperacillin, amoxicillin+clavulanic acid, ceftazidime, ampicillin+clavulanic acid, amoxicillin, ampicillin	DQ112222
		aac(6')-Il	tobramycin, amikacin	U13880
		qacE	cetylpyridinium chloride, chlorhexidine, benzylkonium chloride, ethidium bromide	X68232
		aadA6	spectinomycin, streptomycin	AF140629
		sul1	sulfamethoxazole	U12338
		aac (6')-31	netilmicin, sisomicin, neomycin, tobramycin, isepamicin, gentamicin, amikacin, kanamycin	AM283489
		catB7	chloramphenicol	AF036933
	ISPa7 Composite transposon	*aadA6*	spectinomycin, streptomycin	AF140629
		sul1	sulfamethoxazole	U12338
		aac(6')-31	netilmicin, sisomicin, neomycin, tobramycin, isepamicin, gentamicin, amikacin, kanamycin	AM283489
		blaOXA-2	piperacillin, amoxicillin+clavulanic acid, ceftazidime, ampicillin+clavulanic acid, amoxicillin, ampicillin	DQ112222
		aac(6')-Il	tobramycin, amikacin	U13880
		qacE	cetylpyridinium chloride, chlorhexidine, benzylkonium chloride, ethidium bromide	X68232
	ISPa7 Composite transposon	*aadA6*	spectinomycin, streptomycin	AF140629
		sul1	sulfamethoxazole	U12338
		aac(6')-31	netilmicin, sisomicin, neomycin, tobramycin, isepamicin, gentamicin, amikacin, kanamycin	AM283489
		blaOXA-2	piperacillin, amoxicillin+clavulanic acid, ceftazidime, ampicillin+clavulanic acid, amoxicillin, ampicillin	DQ112222
		aac(6')-Il	tobramycin, amikacin	U13880
		qacE	cetylpyridinium chloride, chlorhexidine, benzylkonium chloride, ethidium bromide	X68232
**Isolate 1607533**				
234	IS6100 Composite transposon	*fosA*	fosfomycin	ACWU01000146
		aph(3'')-Ib	streptomycin	AF024602
		aph(6)-Id	streptomycin	M28829

		catB7	chloramphenicol	AF036933
		crpP	ciprofloxacin	HM560971
	Tn5563<br>Unit transposon	*qacE*	ethidium bromide, chlorhexidine, cetylpyridinium chloride, benzylkonium chloride	X68232
		ant(2'')-Ia	gentamicin, tobramycin	X04555
		sul1	sulfamethoxazole	X15024
		blaOXA-2	amoxicillin, piperacillin, amoxicillin+clavulanic acid, ampicillin+clavulanic acid, ceftazidime, ampicillin	DQ112222
**Isolate 1690076**				
309	Tn5563<br>Unit transposon	*fosA*	fosfomycin	ACWU01000146
		aac(6')-33	tobramycin, amikacin	GQ337064
		qacE	chlorhexidine, cetylpyridinium chloride, ethidium bromide, benzylkonium chloride	X68232
		sul1	sulfamethoxazole	X15024
		aac(6')-Ib-cr	tobramycin, amikacin, ciprofloxacin	EF636461
		blaGES-19	ticarcillin, piperacillin, cefoxitin, ceftazidime, ampicillin+clavulanic acid, amoxicillin, ampicillin, amoxicillin+clavulanic acid	JN596280
		blaOXA-2	piperacillin, ceftazidime, ampicillin+clavulanic acid, amoxicillin, ampicillin, amoxicillin+clavulanic acid	DQ112222
		aac(6')-Ib3	tobramycin, amikacin	X60321
		catB7	chloramphenicol	AF036933
		ant(2”)-Ia	gentamicin, tobramycin	M95287
		blaGES-20	ticarcillin, piperacillin, cefoxitin, ceftazidime, ampicillin+clavulanic acid, amoxicillin, ampicillin, amoxicillin+clavulanic acid	JN596280
	IS6100<br>Composite transposon	*tet(G)*	tetracycline, doxycycline	AF133139

[a] Transposable elements were identified using MobileElementFinder v1.0.3.

Table S4. MICs for aminoglycosides of *P. aeruginosa* containing pSW196[a]

**Isolate**	**Arabinose absent**			**Arabinose present**		
	Tob	Gen	Amik	Tob	Gen	Amik
**Laboratory reference strain**						
PAO1	0.5	1	2	0.5	1	2
PAO1 [pSW196]	0.5	1	2	0.5	1	2
PAO1Δ*mexXY*	0.25	0.25	0.5	0.25	0.25	0.5
PAO1Δ*mexXY* [pSW196]	0.25	0.25	0.5	0.25	0.25	0.5
PAO1Δ*mexZ*	1	2	4	1	2	4
PAO1Δ*mexZ* [pSW196]	1	2	4	1	2	4
PAO1*fusA1*$_{R680C}$	2	4	8	2	4	8
PAO1*fusA1*$_{R680C}$ [pSW196]	2	4	8	2	4	8
**Clinical Strains**						
1257147	512	256	512	512	256	512
1257147 [pSW196]	512	256	512	512	256	512
1260990	32	64	128	32	64	128
1260990 [pSW196]	32	64	128	32	64	128
006A2	32	32	128	32	32	128
006A2 [pSW196]	32	32	128	32	32	128
403-107	2	4	16	2	4	16
403-107 [pSW196]	2	4	16	2	4	16
015A	8	16	32	8	16	32
015A [pSW196]	8	16	32	8	16	32

a MICs for tobramycin (Tob), gentamicin (Gen) and amikacin (Amik) were determined in triplicate, as described in the main text, and median values are shown.

Table S5. Bacterial isolates and plasmids used in this study.

Strain	Source	Patient Location/Sample Information	Bio Sample	MLST Type	Reference
***P. aeruginosa* reference strain**					
PAO1			SAMN11606715	549	[26]
**Clinical isolates of *P. aeruginosa***					
015A	Adult CF	Dunedin	SAMN07424137	499	[27,28]
403-105	Adult CF	Brisbane	SAMN24255275	775	[27]
403-107	Adult CF	Brisbane	SAMN24255277	17	[27]
006-A2	Child CF	Brisbane	SAMN11606760	821	[27,28]
008-A1	Adult CF	Brisbane	SAMN11606756	775	[27]
1257147	Bladder	Argentina	SAMN15663255	235	[29]
1260990	Urine	Greece	SAMN15663259	395	[29]
1268230	Wound	Spain	SAMN15663262	175	[29]
1271701	Urine	Israel	SAMN15663264	1560	[29]
1275655	Wound	Mexico	SAMN15663267	235	[29]
1295835	Sputum	Italy	SAMN15663273	646	[29]
1324459	Burn	Romania	SAMN15663277	357	[29]
1344658	Respiratory	Turkey	SAMN15663278	292	[29]
1420275	Respiratory	Mexico	SAMN15663281	309	[29]
1586994	Blood	Belgium	SAMN15663287	235	[29]
1607533	Colon	Russia	SAMN15663288	234	[29]
1690076	Respiratory	Mexico	SAMN15663290	309	[29]
***P. aeruginosa* engineered mutants**					
**Mutants**	**Genotype/ relevant characteristics**				**Reference**
PAO1Δ *mexXY*	Deletion of *mexXY* genes in reference strain PAO1				[30]
PAO1Δ *mexZ*	Deletion of *mexZ* gene in reference strain PAO1				[30]
PAO1 $fusA1_{R680C}$	R680C mutation in elongation factor G *fusA1* of reference strain PAO1				This study
015AΔ *mexXY*	Deletion of *mexXY* genes from isolate 015A				[30]
403-107Δ *mexXY*	Deletion of *mexXY* genes from isolate 403-107				[30]
1257147Δ *mexXY*	Deletion of *mexXY* genes from isolate 1257147				[30]
1260990Δ *mexXY*	Deletion of *mexXY* genes from isolate 1260990				[30]
1257147 Δ *ant (2")-Ia*	Deletion of aminoglycoside nucleotidyltransferases (*ant (2")-Ia*) from isolate 1257147				This study
1260990Δ *aac (6')-Ib3*	Deletion of aminoglycoside acetyltransferases (*aac (6')-Ib3)* from isolate 1260990				This study
1260990Δ *aph (3')-VIa*	Deletion of aminoglycoside phosphoryltransferases (*aph (3')-VIa*) from isolate 1260990				This study
1260990Δ *aac (6')-Ib3* Δ *aph (3')-VIa*	Deletion of aminoglycoside acetyltransferases (*aac (6')-Ib3*) and aminoglycoside phosphoryltransferases (*aph (3')-VIa)* from isolate 1260990				This study

***P. aeruginosa* carrying cloned genes encoding aminoglycoside modifying enzymes**		
**Strains**	**Genotype/ relevant characteristics**	**Reference**
PAO1 [pSW196]	Strain PAO1 with pSW196 vector integrated at chromosomal *attB* site	This study
PAO1 [pSW196::*ant (2")-Ia*]	Strain PAO1 expressing *ant (2")-Ia*	This study
PAO1 [pSW196::*aac (6')-Ib3*]	Strain PAO1 expressing *aac (6')-Ib3*	This study
PAO1 [pSW196- *aph (3')-VIa*]	Strain PAO1 expressing *aph (3')-VIa*	This study
PAO1Δ *mexXY* [pSW196]	PAO1Δ *mexXY* with pSW196 vector integrated at chromosomal *attB* site	This study
PAO1Δ*mexXY* [pSW196::*ant (2")-Ia*]	PAO1Δ *mexXY* expressing *ant (2")-Ia*	This study
PAO1Δ*mexXY* [pSW196::*aac (6')-Ib3*]	PAO1Δ *mexXY* expressing *aac (6')-Ib3*	This study
PAO1Δ*mexXY* [pSW196:: *aph (3')-VIa*]	PAO1Δ *mexXY* expressing *aph (3')-VIa*	This study
PAO1Δ *mexZ* [pSW196]	PAO1Δ *mexZ* with pSW196 vector integrated at chromosomal *attB* site	This study
PAO1Δ*mexZ* [pSW196::*ant (2")-Ia*]	PAO1Δ *mexZ* expressing *ant (2")-Ia*	This study
PAO1Δ*mexZ* [pSW196::*aac (6')-Ib3*]	PAO1Δ *mexZ* expressing *aac (6')-Ib3*	This study
PAO1Δ*mexZ* [pSW196:: *aph (3')-VIa*]	PAO1Δ *mexZ* expressing *aph (3')-VIa*	This study
PAO1 $fusA1_{\mathrm{R680C}}$ [pSW196]	PAO1 $fusA1_{\mathrm{R680C}}$ with pSW196 vector integrated at chromosomal *attB* site	This study
PAO1$fusA1_{\mathrm{R680C}}$ [pSW196::*ant (2")-Ia*]	PAO1 $fusA1_{\mathrm{R680C}}$ expressing *ant (2")-Ia*	This study
PAO1$fusA1_{\mathrm{R680C}}$ [pSW196::*aac (6')-Ib3*]	PAO1 $\mathrm{fusA1}_{\mathrm{R680C}}$ expressing *aac (6')-Ib3*	This study
PAO1$fusA1_{\mathrm{R680C}}$ [pSW196:: *aph (3')-VIa*]	PAO1 $\mathrm{fusA1}_{\mathrm{R680C}}$ expressing *aph (3')-VIa*	This study
006A2 [pSW196]	006A2 with pSW196 vector integrated at chromosomal *attB* site	This study
006A2 [pSW196::*ant (2")-Ia*]	006A2 expressing *ant (2")-Ia*	This study
006A2 [pSW196::*aac (6')-Ib3*]	006A2 expressing *aac (6')-Ib3*	This study
006A2 [pSW196::*aph (3')-VIa*]	006A2 expressing *aph (3')-VIa*	This study
006A2Δ*mexXY* [pSW196]	006A2Δ*mexXY* with pSW196 vector integrated at chromosomal *attB* site	This study

006A2Δ*mexXY* [pSW196::*ant (2")-Ia*]	006A2Δ*mexXY* expressing *ant (2")-Ia*	This study
006A2Δ*mexXY* [pSW196::*aac (6')-Ib3*]	006A2Δ*mexXY* expressing *aac (6')-Ib3*	This study
006A2Δ*mexXY* [pSW196:: *aph (3')-VIa*]	006A2Δ*mexXY* expressing *aph (3')-VIa*	This study
403-107 [pSW196]	403-107 with pSW196 vector integrated at chromosomal *attB* site	This study
403-107 [pSW196::*ant (2")-Ia*]	403-107 expressing *ant (2")-Ia*	This study
403-107 [pSW196::*aac (6')-Ib3*]	403-107 expressing *aac (6')-Ib3*	This study
403-107 [pSW196::*aph (3')-VIa*]	403-107 expressing *aph (3')-VIa*	This study
403-107Δ*mexXY* [pSW196]	403-107Δ*mexXY* with pSW196 vector integrated at chromosomal attB site	This study
403-107Δ*mexXY* [pSW196 *::ant (2")-Ia*]	403-107Δ*mexXY* expressing *ant (2")-Ia*	This study
403-107Δ*mexXY* [pSW196 *::aac (6')-Ib3*]	403-107Δ*mexXY* expressing *aac (6')-Ib3*	This study
403-107Δ*mexXY* [pSW196 ::*aph (3')-VIa*]	403-107Δ*mexXY* expressing *aph (3')-VIa*	This study
015A [pSW196]	015A with pSW196 vector integrated at chromosomal *attB* site	This study
015A [pSW196::*ant (2")-Ia*]	015A expressing *ant (2")-Ia*	This study
015A [pSW196::*aac (6')-Ib3*]	015A expressing *aac (6')-Ib3*	This study
015A [pSW196::*aph (3')-VIa*]	015A expressing *aph (3')-VIa*	This study
015AΔ*mexXY* [pSW196]	015AΔ*mexXY* with pSW196 vector integrated at chromosomal *attB* site	This study
015AΔ*mexXY* [pSW196::*ant (2")-Ia*]	015AΔ*mexXY* expressing *ant (2")-Ia*	This study
015AΔ*mexXY* [pSW196::*aac (6')-Ib3*]	015AΔ*mexXY* expressing *aac (6')-Ib3*	This study
015AΔ*mexXY* [pSW196::*aph (3')-VIa*]	015AΔ*mexXY* expressing *aph (3')-VIa*	This study
1257147Δ *ant (2")-Ia* [pSW196::*ant (2")-Ia*]	1257147Δ *ant (2")-Ia* expressing *ant (2")-Ia*	This study
1260990Δ *aac (6')-Ib3* [pSW196::*aac (6')-Ib3*]	1260990Δ *aac (6')-Ib3* expressing *aac (6')-Ib3*	This study
1260990Δ *aph (3')-VIa* [pSW196::*aph (3')-VIa*]	1260990Δ *aph (3')-VIa* expressing *aph (3')-VIa*	This study

**Strains of *Escherichia coli***		
**Strain**	**Genotype/ relevant characteristics**	**Reference**
JM83	*ara*, Δ(*lac-proAB*) *rpsL*(*strA*), φ80, *lacZ*ΔM15	[31]
ST18	S 17 λ*pir*Δ*hemA*	[32]
**Plasmids used in the study**		
**Plasmids**	**Genotype/ relevant characteristics**	**Reference**
pEX18Tc	pMB1 replicon, oriT[a] sacB[b], Tc^{R} [c], allelic exchange vector	[33]
pEX18Tc::Δ*mexXY*	pEX18Tc derivatives carrying *mexXY* (*PA2018-PA2019*) flanking regions	This study
pEX18Tc::Δ*mexZ*	pEX18Tc derivatives carrying *mexZ* (*PA2020*) flanking regions	This study
pEX18Tc::*fusA1*(R680C)	pEX18Tc	This study
pEX18Tc::Δ ant (2")-Ia	pEX18Tc derivatives carrying ant (2")-Ia flanking regions	This study
pEX18Tc::Δ aac (6')-Ib3	pEX18Tc derivatives carrying aac (6')-Ib3 flanking regions	This study
pEX18Tc::Δ aph (3')-VIa	pEX18Tc derivatives carrying aph (3')-VIa flanking regions	This study
pSW196	Site-specific integrative plasmid (*attB* site), *araC*-pBAD cassette	[34]
pSW196::ant (2")-Ia	pSW196 expressing arabinose-inducible *ant (2")-Ia*	This study
pSW196::aac (6')-Ib3	pSW196 expressing arabinose-inducible *aac (6')-Ib3*	This study
pSW196::aph (3')-VIa	pSW196 expressing arabinose-inducible *aph (3')-VIa*	This study

Supplementary Table S6. Primers used in this study.

**Primers**	**Sequences[a]**
Ant (2)-Ia_F	5' CCCCC GAATTC GTGGCGGTTTTCATGGCTT 3'
Ant (2)-Ia_R	5' GGGGG GCGGCCGC GCTTGGACGAATTGTTAGGC 3'
Ant_F1F	5' CCCCC GAATTC GTGGCGGTTTTCATGGCTT 3'
Ant_F1R	5' GGGGG TCTAGA AAGCAGGTTCGCAGTCAAGT 3'
Ant_F2F	5' CCCCC TCTAGA CCGCTTTCAGGTCGCGATA 3'
Ant_F2R	5' GGGGG AAGCTT GAAACTTGTATAGCGCCCCTTA 3'
Ant_SF	5' GATGCGTGGAGACCGAAAC 3'
Ant_SR	5' ACCATGCACTGATTCACTGG 3'
Aac (6)-Ib_F	5' CCCCC GAATTC GCTGGACAGTCCCAGTCG 3'
Aac (6)-Ib_R	5' GGGGG GCGGCCGC GATGGAAGGGTTAGGCATCA 3'
Aac_F1F	5' CCCCC GAATTC CAGCGAGCCATTTGAGTCAA 3'
Aac_F1R	5' GGGGG GGTACC GGAATCGTTGCTGTTGGTCA 3'
Aac_F2F	5' CCCCC GGTACC TGCCTAACCCTTCCATCGAG 3'
Aac_F2R	5' GGGGG AAGCTT CATCGTGATCTCCGTTCGTG 3'
Aac_SF	5' TCTGGTGTGGGAACTGAGTC 3'
Aac_SR	5' CCGGAATTTCGCTGACTGTC 3'
Aph (3')-VIa_CF	5' GGGG GCGGCCGC TCATCTATTTACTAGGCCTCGCA 3'
Aph (3')-VIa_CR	5' GGGGG GAGCTC GGTGGTTTATGTCGCACTTCA 3'
Aph_F1_F	5' CCCCC GAGCTC TGCCGCGAATGGTATTGAC 3'
Aph_FI_R	5' CCCCC TCTAGA GAGAGGATGCATCGGAGGAA 3'
Aph_F2_F	5' CCCCC TCTAGA TGGCTCTAAAACGCTGTTTCC 3'
Aph_F2_R1	5' CCCCC AAGCTT GTATCAGCTCAAGACGCCGT 3'
Aph_SR_F	5' CGCACTTCAAGTTTTACTCTGC 3'
Aph_SR_R	5' TGGCTTTTGAAACTGTCGCA 3'
*mexXY*_F1	5' CCCCC GGTACC GAGTCGGCTGATG ACCTACA 3'
*mexXY*_R1	5'GGGGG TCTAGA GTACCGCTGTTCTTCCTGGT 3'
*mexXY*_F2	5' CCCCC TCTAGATGTCCCTCGATTC GTGAACT 3'
*mexXY*_R2	5' GGGGG AAGCTT GCTCTACATCGAC GGCAAG 3'
*PA2017*_F	5' GCA GCC TGT ACG TGG TCA 3'
*mexZ*_R	5' GGG TTT TCT GGG ATT CCT CT 3'
*mexX*_F	5' CCCCC GAATTC GTTCTCGACGATCACCCACT 3'
*mexX*_R	5' GGGGG TCTAGA GGGTTTTCTGGGATTCCTCT 3'
*PA2022*_F	5' CCCCC TCTAGA CGCAGTTCTCCCTACCTGTT 3'
*PA2022*_R	5' GGGGG AAGCTT CGCAGTATCTGGCTGTCGTA 3'
*mexZ*_SR_F	5' GTGTCCCTCGATTCGTGAAC 3'

*mexZ*_SR_R	5' CGTGAAGCTACCGTGACAGA 3'
fusA1_F	5' GGGGG TCTAGA TTACTCGATGATCTTGGCAAC 3'
fusA1_R	5' CCCCC AAGCTT GAAGCCGAGATCAAGGAAGG 3'
*fusA1*_Scr_F	5' GTATTCAACGTGCGAGGTGT 3'
*fusA1*_Scr_R	5' GTTCAAGATCGCTGCTTCCA 3'
CTXmcsDown	5' TGACTACGTGGTTCCTGGCCTG
CTXmcsUP	5' TTCAAAAGGTCATCCACCGGC

[a]Introduced restriction sites are underlined.

References are listed below.

1. Yoshii, A.; Moriyama, H.; Fukuhara, T. The novel kasugamycin 2′-N-acetyltransferase gene aac (2′)-IIa, carried by the IncP island, confers kasugamycin resistance to rice-pathogenic bacteria. *Appl Environ Microbiol.* **2012**, *78*, 5555-5564.
2. Haines, A.S.; Jones, K.; Cheung, M.; Thomas, C.M. The IncP-6 plasmid Rms149 consists of a small mobilizable backbone with multiple large insertions. *J Bacteriol.* **2005**, *187*, 4728-4738.
3. Gibb, A.P.; Tribuddharat, C.; Moore, R.A.; Louie, T.J.; Krulicki, W.; Livermore, D.M.; Palepou, M.-F.I.; Woodford, N. Nosocomial outbreak of carbapenem-resistant *Pseudomonas aeruginosa* with a new bla IMP allele, bla IMP-7. *Antimicrob Agents Chemother.* **2002**, *46*, 255-258.
4. Tenover, F.C.; Phillips, K.; Gilbert, T.; Lockhart, P.; O'Hara, P.; Plorde, J. Development of a DNA probe from the deoxyribonucleotide sequence of a 3-N-aminoglycoside acetyltransferase [AAC (3)-I] resistance gene. *Antimicrob Agents Chemother.* **1989**, *33*, 551-559.
5. MacLeod, D.L.; Nelson, L.E.; Shawar, R.M.; Lin, B.B.; Lockwood, L.G.; Dirks, J.E.; Miller, G.H.; Burns, J.L.; Garber, R.L. Aminoglycoside-resistance mechanisms for cystic fibrosis *Pseudomonas aeruginosa* isolates are unchanged by long-term, intermittent, inhaled tobramycin treatment. *J Infect Dis.* **2000**, *181*, 1180-1184.
6. Vliegenthart, J.; Ketelaar-van Gaalen, P.; van de Klundert, J. Nucleotide sequence of the aacC3 gene, a gentamicin resistance determinant encoding aminoglycoside-(3)-N-acetyltransferase III expressed in *Pseudomonas aeruginosa* but not in *Escherichia coli*. *Antimicrob Agents Chemother.* **1991**, *35*, 892-897.
7. Plattner, M.; Gysin, M.; Haldimann, K.; Becker, K.; Hobbie, S.N. Epidemiologic, phenotypic, and structural characterization of aminoglycoside-resistance gene aac (3)-IV. *Int J Mol Sci.* **2020**, *21*, 6133.
8. Casin, I.; Bordon, F.; Bertin, P.; Coutrot, A.; Podglajen, I.; Brasseur, R.; Collatz, E. Aminoglycoside 6′-N-acetyltransferase variants of the Ib type with altered substrate profile in clinical isolates of *Enterobacter cloacae* and *Citrobacter freundii*. *Antimicrob Agents Chemother.* **1998**, *42*, 209-215.
9. Rather, P.; Munayyer, H.; Mann, P.; Hare, R.; Miller, G.; Shaw, K. Genetic analysis of bacterial acetyltransferases: identification of amino acids determining the specificities of the aminoglycoside 6'-N-acetyltransferase Ib and IIa proteins. *J Bacteriol.* **1992**, *174*, 3196-3203.
10. Poole, K. Aminoglycoside resistance in *Pseudomonas aeruginosa*. *Antimicrob Agents Chemother.* **2005**, *49*, 479-487.
11. Mendes, R.E.; Castanheira, M.; Toleman, M.A.; Sader, H.S.; Jones, R.N.; Walsh, T.R. Characterization of an integron carrying blaIMP-1 and a new aminoglycoside resistance gene, aac (6′)-31, and its dissemination among genetically unrelated clinical isolates in a Brazilian hospital. *Antimicrob Agents Chemother.* **2007**, *51*, 2611-2614.
12. Viedma, E.; Juan, C.; Acosta, J.; Zamorano, L.; Otero, J.R.; Sanz, F.; Chaves, F.; Oliver, A. Nosocomial spread of colistin-only-sensitive sequence type 235 *Pseudomonas aeruginosa* isolates producing the extended-spectrum β-lactamases GES-1 and GES-5 in Spain. *Antimicrob Agents Chemother.* **2009**, *53*, 4930-4933.
13. Hollingshead, S.; Vapnek, D. Nucleotide sequence analysis of a gene encoding a streptomycin/spectinomycin adenyltransferase. *Plasmid.* **1985**, *13*, 17-30.
14. Gu, B.; Tong, M.; Zhao, W.; Liu, G.; Ning, M.; Pan, S.; Zhao, W. Prevalence and characterization of class I integrons among *Pseudomonas aeruginosa* and *Acinetobacter baumannii* isolates from patients in Nanjing, China. *J Clin Microbiol.* **2007**, *45*, 241-243.
15. Kazama, H.; Kizu, K.; Iwasaki, M.; Hamashima, H.; Sasatsu, M.; Arai, T. A new gene, aadA2b, encoding an aminoglycoside adenylyltransferase, AAD (3")(9), isolated from integron InC in *Pseudomonas aeruginosa*. *Microbios.* **1996**, *86*, 77-83.

16. Adrian, P.V.; Thomson, C.J.; Klugman, K.P.; Amyes, S.G. New gene cassettes for trimethoprim resistance, dfr13, and streptomycin-spectinomycin resistance, aadA4, inserted on a class 1 integron. *Antimicrob Agents Chemother.* **2000**, *44*, 355-361.
17. Papadovasilaki, M.; Oberthür, D.; Gessmann, R.; Sarrou, I.; Betzel, C.; Scoulica, E.; Petratos, K. Biophysical and enzymatic properties of aminoglycoside adenylyltransferase AadA6 from *Pseudomonas aeruginosa*. *Biochem Biophys Rep.* **2015**, *4*, 152-157.
18. Santanam, P.; Kayser, F.H. Purification and characterization of an aminoglycoside inactivating enzyme from *Staphylococcus epidermidis* FK109 that nucleotidylates the 4'-and 4"-hydroxyl groups of the aminoglycoside antibiotics. *J Antibiot.* **1978**, *31*, 343-351.
19. Esparragón, F.R.; Martín, M.G.; Lama, Z.G.; Sabatelli, F.J.; Junco, M.T.T. Aminoglycoside resistance mechanisms in clinical isolates of *Pseudomonas aeruginosa* from the Canary Islands. *Zentralbl Bakteriol.* **2000**, *289*, 817-826.
20. Woegerbauer, M.; Kuffner, M.; Domingues, S.; Nielsen, K.M. Involvement of aph (3')-IIa in the formation of mosaic aminoglycoside resistance genes in natural environments. *Front Microbiol.* **2015**, *6*, 442.
21. Lambert, T.; Gerbaud, G.; Courvalin, P. Characterization of transposon Tn1528, which confers amikacin resistance by synthesis of aminoglycoside 3'-O-phosphotransferase type VI. *Antimicrob Agents Chemother.* **1994**, *38*, 702-706.
22. Stogios, P.J.; Shakya, T.; Evdokimova, E.; Savchenko, A.; Wright, G.D. Structure and function of APH (4)-Ia, a hygromycin B resistance enzyme. *J Biol Chem.* **2011**, *286*, 1966-1975.
23. Ashenafi, M.; Ammosova, T.; Nekhai, S.; Byrnes, W.M. Purification and characterization of aminoglycoside phosphotransferase APH (6)-Id, a streptomycin-inactivating enzyme. *Mol Cell Biochem.* **2014**, *387*, 207-216.
24. Bortolaia, V.; Kaas, R.S.; Ruppe, E.; Roberts, M.C.; Schwarz, S.; Cattoir, V.; Philippon, A.; Allesoe, R.L.; Rebelo, A.R.; Florensa, A.F. ResFinder 4.0 for predictions of phenotypes from genotypes. *J Antimicrob Chemother.* **2020**, *75*, 3491-3500.
25. Alcock, B.P.; Raphenya, A.R.; Lau, T.T.; Tsang, K.K.; Bouchard, M.; Edalatmand, A.; Huynh, W.; Nguyen, A.-L.V.; Cheng, A.A.; Liu, S. CARD 2020: antibiotic resistome surveillance with the comprehensive antibiotic resistance database. *Nucleic Acids Res.* **2020**, *48*, D517-D525.
26. Stover, C.K.; Pham, X.Q.; Erwin, A.; Mizoguchi, S.; Warrener, P.; Hickey, M.; Brinkman, F.; Hufnagle, W.; Kowalik, D.; Lagrou, M. Complete genome sequence of *Pseudomonas aeruginosa* PAO1, an opportunistic pathogen. *Nature.* **2000**, *406*, 959-964.
27. Martin, L.W.; Robson, C.L.; Watts, A.M.; Gray, A.R.; Wainwright, C.E.; Bell, S.C.; Ramsay, K.A.; Kidd, T.J.; Reid, D.W.; Brockway, B. Expression of *Pseudomonas aeruginosa* antibiotic resistance genes varies greatly during infections in cystic fibrosis patients. *Antimicrob Agents Chemother.* **2018**, *62*, e01789-01718.
28. Wardell, S.J.; Rehman, A.; Martin, L.W.; Winstanley, C.; Patrick, W.M.; Lamont, I.L. A large-scale whole-genome comparison shows that experimental evolution in response to antibiotics predicts changes in naturally evolved clinical *Pseudomonas aeruginosa*. *Antimicrob Agents Chemother.* **2019**, *63*, e01619-01619.
29. Rehman, A.; Jeukens, J.; Levesque, R.C.; Lamont, I.L. Gene-gene interactions dictate ciprofloxacin resistance in *Pseudomonas aeruginosa* and facilitate prediction of resistance phenotype from genome sequence data. *Antimicrob Agents Chemother.* **2021**, AAC. 02696-02620.
30. Thacharodi, A.; Lamont, I.L. Aminoglycoside resistance in *Pseudomonas aeruginosa*: the contribution of the MexXY-OprM efflux pump varies between isolates. *J Med Microbiol* **2022**, *(In press)*.
31. Yanisch-Perron, C.; Vieira, J.; Messing, J. Improved M13 phage cloning vectors and host strains: nucleotide sequences of the M13mpl8 and pUC19 vectors. *Gene.* **1985**, *33*, 103-119.

32. Thoma, S.; Schobert, M. An improved *Escherichia coli* donor strain for diparental mating. *FEMS Microbiol Lett.* **2009**, *294*, 127-132.
33. Hoang, T.T.; Karkhoff-Schweizer, R.R.; Kutchma, A.J.; Schweizer, H.P. A broad-host-range Flp-FRT recombination system for site-specific excision of chromosomally-located DNA sequences: application for isolation of unmarked *Pseudomonas aeruginosa* mutants. *Gene.* **1998**, *212*, 77-86.
34. Baynham, P.J.; Ramsey, D.M.; Gvozdyev, B.V.; Cordonnier, E.M.; Wozniak, D.J. The *Pseudomonas aeruginosa* ribbon-helix-helix DNA-binding protein AlgZ (AmrZ) controls twitching motility and biogenesis of type IV pili. *J Bacteriol.* **2006**, *188*, 132-140, doi:10.1128/JB.188.1.132-140.2006.
